# Transection of the cervical sympathetic trunk inhibits the progression of pulmonary arterial hypertension via ERK-1/2 Signalling

**DOI:** 10.1186/s12931-019-1090-2

**Published:** 2019-06-14

**Authors:** Yongpeng Zhao, Rui Xiang, Xin Peng, Qian Dong, Dan Li, Guiquan Yu, Lei Xiao, Shu Qin, Wei Huang

**Affiliations:** 1 Department of Cardiology, the First Affiliated Hospital of Chongqing Medical University, 1 Youyi Road, Yuzhong District, Chongqing, China; 2 Department of Medicine, Section of Pulmonary, Critical Care, Sleep and Allergy, University of Illinois at Chicago, Chicago, IL USA; 3 Present Address: Lung Vascular Biology Program, NHLBI/NIH, Bethesda, MD USA

**Keywords:** Transection of the cervical sympathetic trunk, Sympathetic nerve block, Pulmonary arterial hypertension

## Abstract

**Background:**

Abnormal sympathetic hyperactivity has been shown to lead to pulmonary arterial hypertension (PAH) deterioration. The purpose of this study was to examine whether the transection of the cervical sympathetic trunk (TCST) can inhibit the progression of PAH in a monocrotaline (MCT)-induced PAH model and elucidate the underlying mechanisms.

**Methods:**

Rats were randomly divided into four groups, including a control group, an MCT group, an MCT + sham group and an MCT + TCST group. After performing haemodynamic and echocardiographic measurements, the rats were sacrificed for the histological study, and the norepinephrine (NE) concentrations and protein expression level of tyrosine hydroxylase (TH) were evaluated. The protein expression levels of extracellular signal-regulated kinase (ERK)-1/2, proliferating cell nuclear antigen (PCNA), cyclin A2 and cyclin D1 in pulmonary artery vessels and pulmonary arterial smooth muscle cells (PASMCs) were determined.

**Results:**

Compared with the MCT + sham group, TCST profoundly reduced the mean pulmonary arterial pressure (mPAP) (22.02 ± 4.03 mmHg vs. 31.71 ± 2.94 mmHg), right ventricular systolic pressure (RVSP) (35.21 ± 5.59 mmHg vs. 48.36 ± 5.44 mmHg), medial wall thickness (WT%) (22.48 ± 1.75% vs. 46.10 ± 3.16%), and right ventricular transverse diameter (RVTD) (3.78 ± 0.40 mm vs. 4.36 ± 0.29 mm) and increased the tricuspid annular plane systolic excursion (TAPSE) (2.00 ± 0.12 mm vs. 1.41 ± 0.24 mm) (all *P* < 0.05). The NE concentrations and protein expression levels of TH were increased in the PAH rats but significantly decreased after TCST. Furthermore, TCST reduced the increased protein expression of PCNA, cyclin A2 and cyclin D1 induced by MCT in vivo. We also found that NE promoted PASMC viability and activated the ERK-1/2 pathway. However, the abovementioned NE-induced changes could be suppressed by the specific ERK-1/2 inhibitor U0126.

**Conclusion:**

TCST can suppress pulmonary artery remodelling and right heart failure in MCT-induced PAH. The main mechanism may be that TCST decreases the NE concentrations in lung tissues, thereby preventing NE from promoting PASMC proliferation mediated by the ERK-1/2 signalling pathway.

**Electronic supplementary material:**

The online version of this article (10.1186/s12931-019-1090-2) contains supplementary material, which is available to authorized users.

## Background

Pulmonary arterial hypertension (PAH) is a progressive disease, defined as an increase in the mean pulmonary arterial pressure (mPAP) ≥25 mmHg at rest as assessed by right heart catheterization and is associated with a poor prognosis [1]. This disease shares the following common pathophysiological and histological features: pathologic pulmonary vasoconstriction, remodelling of the small pulmonary arteries and thrombosis [2, 3]. These pathological changes contribute to increased pulmonary vascular resistance, ultimately leading to right ventricular (RV) failure and death. While many advances in therapies for PAH have been achieved, the survival rate remains poor (the 1- and 5-year survival rates are 86.3 and 61.2%, respectively) [4, 5].

Over the past two decades, accumulating evidence has suggested that PAH is generally associated with increased sympathetic nervous system activation [6, 7]. In addition, excess sympathetic activation may be an independent predictor of clinical deterioration [7–9]. Therefore, in addition to pharmacological therapy, different treatments, such as renal denervation [10, 11] and pulmonary artery denervation (PADN) [12–15], have been considered to reduce sympathetic activity and improve PAH. Although renal denervation and PADN reportedly decrease mPAP and prevent the progression of PAH in experimental and clinical trials, the mechanism by which denervation acts in the treatment of PAH remains largely unclear. Moreover, there are several limitations to artery denervation as follows: 1) catheter-based radiofrequency denervation of the arterial sympathetic nerves may lead to arterial stenosis; 2) there is no direct measure which can confirm that the renal or pulmonary artery nerves have in fact been denervated; and 3) the denervation procedure may injure vascular parasympathetic nerves. Therefore, investigating new sympathetic blocking methods for the treatment of PAH is essential.

Cervical sympathetic and stellate ganglion blocks (SGBs) have been used for the treatment of several cardiovascular diseases [16–18]. These procedures inhibit sympathetic activity by decreasing efferent cervical and upper thoracic sympathetic outflow, and successful sympathetic blockade can easily be recognized by the presence of Horner’s syndrome [19]. The transection of the cervical sympathetic trunk (TCST) is a procedure leading to long-term and repeated SGBs. Additionally, pulmonary sympathetic innervation mainly originates from the middle and inferior cervical and thoracic sympathetic chain (T2 to T8), and the pulmonary vasculature is the major sympathetic target in the lung [20]. Since most pulmonary sympathetic axons release norepinephrine (NE), we hypothesized that TCST could decrease the production of NE in the lung.

The proliferation of pulmonary artery smooth muscle cells (PASMCs) has been documented to play a critical role during the progression of PAH [3]. Previous studies have demonstrated that NE induces proliferation in PASMCs [21] and contributes to pulmonary vascular remodelling [22]. Moreover, the extracellular signal-regulated kinase 1/2 (ERK-1/2) signalling pathway plays an important role in multiple cellular functions, including differentiation, proliferation and migration [23]. Evidence suggests that the aberrant activation of ERK-1/2 may lead to increased proliferation in PASMCs [21, 24]. Based on the above findings, we hypothesized that TCST could inhibit the progression of PAH. Accordingly, the aims of the present study were to explore 1) whether haemodynamic and pulmonary artery remodelling could be improved by TCST in an MCT-induced PAH model in rats and 2) whether the ERK-1/2 pathway participates in the NE-induced PASMCs proliferation.

## Methods

### Materials

Monocrotaline (MCT, NO. M0418–500), NE (NO.M8550) and prazosin (Pra, NO.MT1050) were provided by Mengbo (Chongqing, China). U0126 (NO. NY-12031) and Cell Counting Kit-8 (CCK-8, NO. HY-K0301) were obtained from MedChemExpress (MCE, NJ, USA). The NE Elisa Kit (NO. 2907A) was obtained from MB-Biology (Jiangsu, China). The anti-tyrosine hydroxylase (TH, NO.#13,106), ERK-1/2 (NO.#4695) and p-ERK-1/2 (NO.#9101) antibodies were provided by Cell Signaling Technology (Beverly, MA, USA). The anti-α-smooth muscle actin (α-SMA) (NO. ab5694), CD3 (NO. ab16669), cyclin A2 (NO. ab181591), and cyclin D1 (NO. ab134175) antibodies were purchased from Abcam (Cambridge, UK). The proliferating cell nuclear antigen (PCNA, NO. 10205–2-AP), β-actin (No.20536–1-AP), and GAPDH (No.10494–1-AP) antibodies and the secondary antibodies (NO.SA00001–10) were obtained from Proteintech (Wuhan, Hubei, China). All reagents were obtained from common commercial sources.

### Animals and experimental design

All animal procedures were carried out in accordance with the ARRIVE guidelines and the UK Animals (Scientific Procedures) Act of 1986 and its associated guidelines. The experimental protocol was approved by the Ethics Committee of Chongqing Medical University (Chongqing, China).

**Fig. 1:**
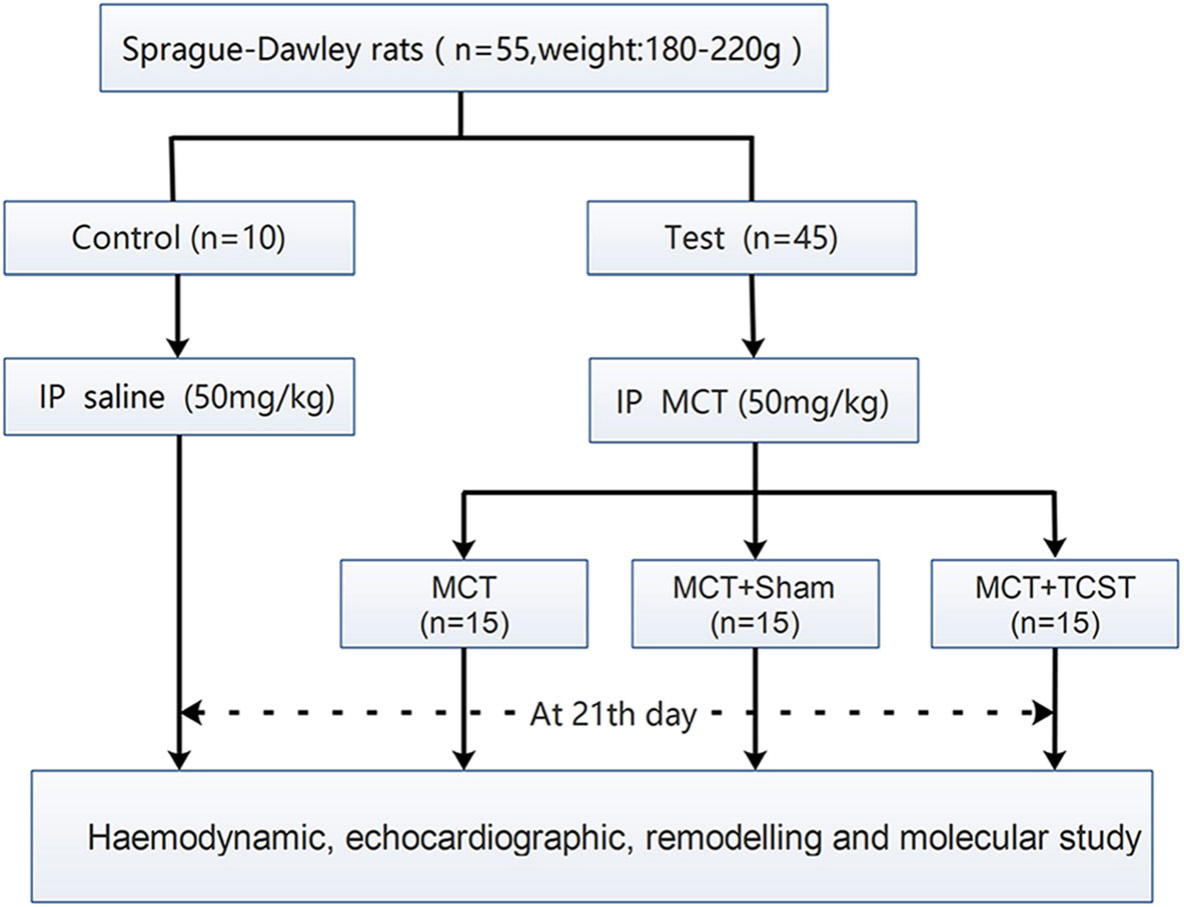
Flow chart of the study. IP, intraperitoneal injection; MCT, monocrotaline; TCST, transection of the cervical sympathetic trunk

The study protocol was shown in Fig. 1. Briefly, fifty-five male Sprague-Dawley rats (aged 8 weeks, weight of 200–250 g) obtained from the Chongqing Laboratory Animal Center were randomly assigned to 4 groups. Group 1 included 10 rats that received intraperitoneal injections of normal saline (Control group). Group 2 included 15 rats that received intraperitoneal injections of MCT (MCT group). Group 3 included 15 rats that received intraperitoneal injections of MCT after 1 h of sham TCST (MCT + sham group). Group 4 included 15 rats that received intraperitoneal injections of MCT after 1 h of TCST (MCT + TCST group). The rats had free access to food and water and were maintained in a room with controlled temperature (22 ± 2 °C) and lighting under 12-h light/dark exposure cycles. At the end of the study, after the haemodynamic parameters were measured and the tissues were collected, all rats were sacrificed.

### MCT-induced PAH

MCT was dissolved in 1 mol/L HCl, the pH was adjusted to 7.4, and the final concentration of MCT was adjusted to 10 mg/ml with distilled water. The MCT-induced PAH rats were intraperitoneally injected with 50 mg/kg of MCT. In the control group, saline was injected instead of MCT.

### Transection of the cervical sympathetic trunk (TCST)

**Fig. 2:**
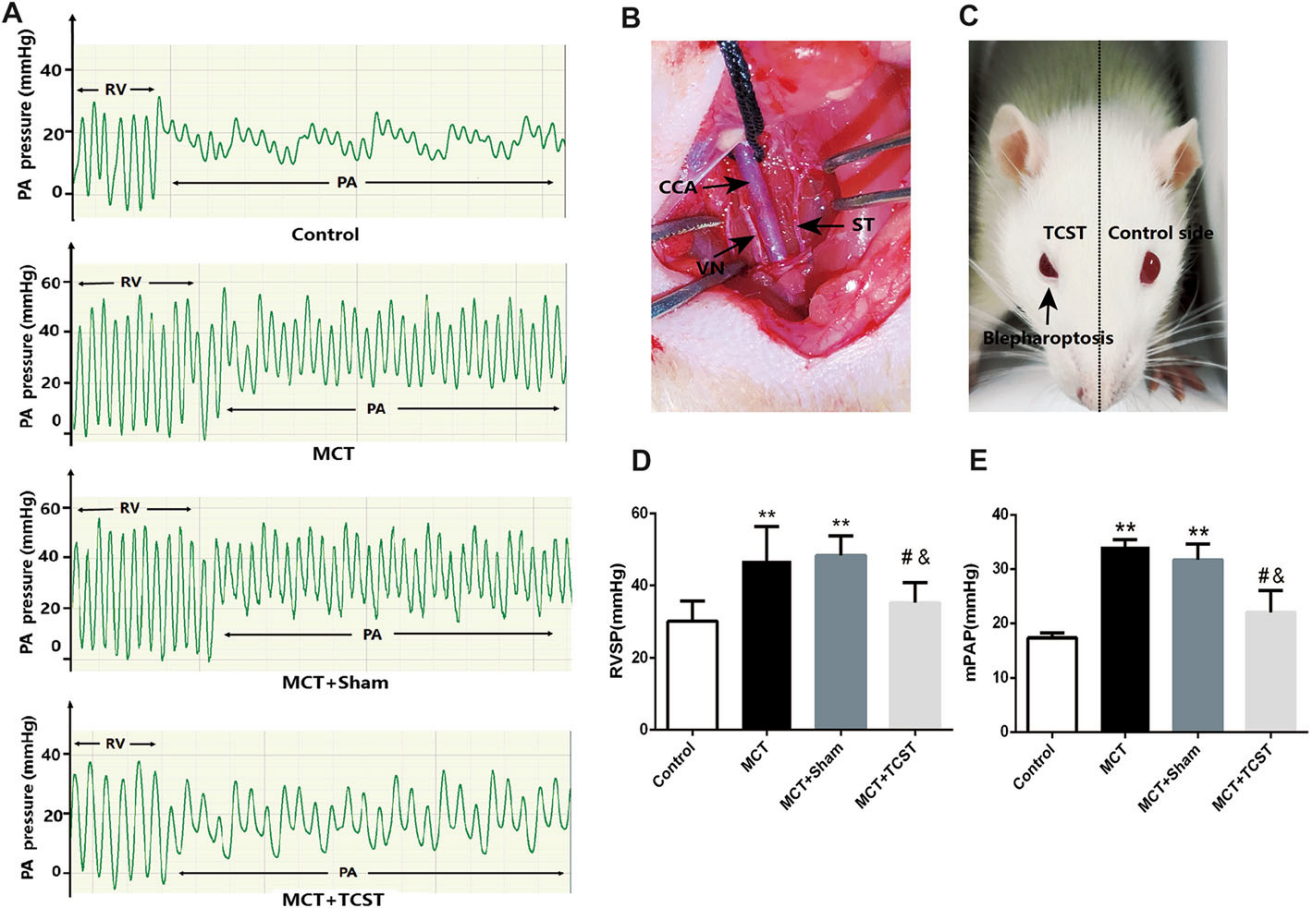
Right ventricular systolic pressure (RVSP) and mean pulmonary arterial pressure (mPAP); (**a**) Assessment of the RVSP and mPAP in MCT-induced pulmonary arterial hypertension (PAH) rats. RV, right ventricle; PA, pulmonary artery. **b** Photograph of the dissection of a rat’s neck. CCA, common carotid artery; VN, vagus nerve; ST, sympathetic trunk. **c** Unilateral blepharoptosis after TCST. **d**, **e** The RVSP and mPAP were significantly elevated in the MCT and MCT + sham groups, whereas this effect was attenuated by TCST. **P < 0.01 vs. control group; #*P* < 0.01 vs. MCT group; &*P* < 0.01 vs. MCT + sham group

Before all surgeries, the rats were anaesthetized with 2% pentobarbital sodium (30 mg/kg, intraperitoneal). A 2-cm vertical incision was created in the neck region. Then, the right sternomastoid muscle and omohyoid muscle were separated (not transected). The right common carotid artery was exposed, and the cervical sympathetic trunk was identified along the proximal side of the superior cervical ganglion [25]. The right side of the cervical sympathetic trunk was transected, and the incision was closed (Fig. 2b). In the rats in the sham group, the right common carotid artery and cervical sympathetic trunk were exposed without transection of the nerve. The sympathetic blockades were confirmed by blepharoptosis on the operated side (Fig. 2c).

### Echocardiographic assessment

Two-dimensional (2D) images of the right ventricular transverse diameter (RVTD) and pulmonary artery transverse diameter (PATD) were measured on apical 4-chamber and parasternal short-axis views, respectively (IE33; Philips, Holland). The left ventricular internal diameter during systole (LVIDs) and left ventricular internal diameter during diastole (LVIDd) were measured on M-mode echocardiography parasternal long-axis views. Then, the cardiac output (CO) was calculated by computer algorithms. The tricuspid annular plane systolic excursion (TAPSE) was measured from M-mode apical 4-chamber views. All data represented the mean of five uninterrupted cardiac cycles.

### Haemodynamic and right ventricular hypertrophy measurements

A homemade polyethylene catheter (inner diameter: 0.5 mm, outside diameter: 0.9 mm) filled with heparin was connected to a Multi-lead Physiological Recorder (MP150, BIOPAC System, USA). Then, the catheter was inserted into the RV of the anaesthetized rats via the right jugular vein and introduced into the pulmonary artery guided by a pressure curve (Fig. 2a). After measuring the pulmonary arterial pressure, the same catheter was inserted into the carotid artery to measure the systemic arterial pressures, and the heart rate (HR) was measured.

To evaluate RV hypertrophy, the atria were removed. Then, the heart was dissected into the RV and left ventricle (LV) plus septal wall and weighed. The ratio of the weight of the RV to the weight of the LV plus septum [RV/(LV + S)] was used as an index of RV hypertrophy.

### Histology and immunohistochemistry

The lung tissues were obtained from anaesthetized rats and immersed in 4% paraformaldehyde overnight for fixation. The fixed tissues were dehydrated, cleared, embedded in paraffin wax, and cut into 5-μm thick sections. Haematoxylin and eosin (H & E), Elastic-Van Gieson, α-SMA and CD3 staining were performed on lung tissue sections.

The immunohistochemical staining procedure was performed according to a previously described method. Briefly, the tissue sections were deparaffinized and rehydrated in graduated alcohol. After the antigen retrieval, the endogenous peroxidase activity was blocked by incubation with 0.3% hydrogen peroxide at room temperature for 10 min. The sections were blocked with 2% bovine serum albumin (BSA) in phosphate-buffered saline (PBS) and then incubated with the α-SMA (1:500) and CD3 (1:200) antibodies overnight, followed by incubation with the secondary antibody. The primary antibody was not applied in the parallel controls. After the colour development through incubation with diaminobenzidine, the sections were counterstained with haematoxylin. The developed tissue sections were imaged under a microscope (Leica Microsystems DFC550, Germany). The CD3 positive index was counted as the percentage of positive cells divided by all lung cells at a high magnification (× 400), and at least 10 independent fields per section were assessed. The wall thickness (WT) and external diameter (ED) of the pulmonary arteries were measured using IPP 6.0 image analysis software (Media Cybernetics). Pulmonary arteriole remodelling was quantified as follows: WT% = 2 x WT/ED × 100%.

### Measurement of NE concentrations

To examine whether MCT induced an increase in the generation of NE, an NE ELISA Kit was used to detect the concentrations of NE in the rat lung homogenate. The samples were thawed on ice, and the concentrations of NE were measured according to the manufacturer’s instructions. The sensitivity of the assay was 8 pg/ml.

### Preparation of PASMCs

Pulmonary artery tissues were isolated from the rats. The pulmonary arteries were minced to a size of 1 × 1 mm, placed in a dish, and incubated with DMEM (Dulbecco’s modified Eagle’s medium) supplemented with 20% foetal bovine serum (FBS), 1% penicillin and streptomycin in a humidified incubator with 5% CO_2_ and 21% O_2_ at 37 °C. The PASMCs were identified as having a smooth muscle phenotype by positive immunofluorescence with an anti-α-SMA antibody. After 3–5 cell passages, the cells were used for the following experiments.

### Cell viability assay

PASMCs (1 × 10^4^ cells/well) were seeded in 96-well plates, covered with 100 μL of medium in each well for 24 h, and subsequently exposed to different treatments in accordance with the group assignment. The cell viability was determined by a Cell Counting Kit-8 and a microplate reader (Multiskan MK33; Thermolab Systems, Helsinki, Finland) at a wavelength of 450 nm.

### EdU staining

A Click-tm EdU Cell Proliferation Kit (NO. C0075S, Beyotime Bio, Shanghai, China) was used for the cell proliferation assay. The PASMCs were incubated with 10 μmol/L EdU for 2 h at 37 °C after treatment. The cells were permeabilized with 0.3% Triton X-100 for 20 min after fixation with 4% formaldehyde for 15 min at room temperature. After three washes with PBS, the cells were incubated with click additive solution for 30 min. PASMC nuclei stained with Hoechst-33,342 were used for the cell counts and examined using fluorescence microscopy. Five randomly selected views per sample image were used to calculate the relative EdU-positive ratio.

### Western blot analysis

Pulmonary artery tissues separated from the lungs of rats were homogenized in ice-cold lysis buffer. The resulting homogenate was centrifuged at 12,000 rpm for 15 min at 4 °C. The supernatants were collected and stored at − 80 °C for the Western blot analysis. The PASMCs were treated with different drugs according to the different experimental groups. The proteins were extracted from the treated PASMCs.

The pulmonary artery tissue homogenates and cell protein samples were separated by SDS-PAGE on a 10–12% gel (No·P0012A, RO Beyotime Bio) and transferred to a PVDF membrane. The membranes were blocked in 5% non-fat milk for 2 h and incubated with the primary antibodies [Beta-actin (1:5000), GAPDH (1:5000) TH (1:1000), ERK-1/2 (1:1000), p-ERK-1/2 (1:1000), PCNA (1:2000), cyclin A2 (1:2000), and cyclin D1 (1:10000)] overnight at 4 °C. The blots were incubated with secondary antibodies (1:5000) for another 2 h at room temperature. The signals were examined with a Bio-Rad gel imaging system (Bio-Rad, Hercules, CA, USA) and a WesternBright™ MCF fluorescent Western blotting kit (Advansta, Menlo Park, USA), and the results were analysed with a gel imaging system.

### Statistical analysis

All experiments were repeated at least three times. The data are reported as the group mean ± standard deviation (SD). SPSS 22.0 (IBM Corporation, Armonk, USA) was used for the statistical analyses. The differences between the groups were determined by Student’s t-tests. A *P*-value < 0.05 was considered statistically significant.

## Results

### Mortality and the PAH model

Twenty-one days after the injection with MCT, in total, 10 deaths occurred, including 6 in the MCT group and 4 in the MCT + sham group. Finally, the control group remained 10 rats, the MCT group remained 9 rats, the MCT + sham group remained 11 rats, and the MCT + TCST group remained 15 rats.

To confirm whether the rat model of PAH established, the mPAP was measured twenty-one days following the MCT injection. Compared with the control rats, the mPAP was significantly increased (Table 1, Fig. 2a and e) in the rats in the MCT group, indicating the successful induction of PAH. There was no significant difference in the mean systemic arterial pressure or HR between the control rats and MCT rats (Table 1), suggesting that MCT had no impact on the systemic arterial pressure.

**Table 1:** Comparison of the haemodynamic data and RV function in the MCT-induced PAH rats

	Control	MCT	MCT + sham	MCT + TCST
HR (beats/min)	408 ± 39	391 ± 24	407 ± 26	421 ± 15
mSAP (mmHg)	108.89 ± 6.88	100.05 ± 7.77	95.08 ± 4.84	108.87 ± 9.30
RVSP (mmHg)	30.85 ± 5.32	46.83 ± 9.53**	48.36 ± 5.44**	35.21 ± 5.59^#&^
mPAP (mmHg)	17.34 ± 0.88	34.13 ± 1.30**	31.71 ± 2.94**	22.02 ± 4.03^#&^
RVTD (mm)	3.69 ± 0.25	4.42 ± 0.27**	4.36 ± 0.29**	3.78 ± 0.40^#&^
PATD (mm)	2.33 ± 0.23	2.43 ± 0.38	2.63 ± 0.26	2.62 ± 0.18
TAPSE (mm)	2.12 ± 0.04	1.34 ± 0.10*	1.41 ± 0.24*	2.00 ± 0.12^$^
CO (ml/min)	199.15 ± 12.17	185.78 ± 12.88**	178.53 ± 6.75**	204.72 ± 11.13^#&^
RV/LV + S	0.22 ± 0.08	0.38 ± 0.08**	0.48 ± 0.10**	0.24 ± 0.07^#&^

All values are expressed as the mean ± SD. CO, cardiac output; HR, heart rate; mPAP, mean pulmonary arterial pressure; RVSP, right ventricular systolic pressure; mSAP, mean systemic arterial pressure; PATD, pulmonary artery transverse diameter; RV/(LV + S), right ventricle/(left ventricle + septum); RVTD, right ventricular transverse diameter; TAPSE, tricuspid annular plane systolic excursion; *P < 0.05 vs. control group; $*P* < 0.05 vs. MCT or MCT + sham group; **P < 0.01 vs. control group; #*P* < 0.01 vs. MCT group; &*P* < 0.01 vs. MCT + sham group

### TCST attenuated the MCT-induced increase in RVSP and mPAP

Twenty-one days after the MCT injections, the RVSP and mPAP in the MCT and MCT + sham groups were significantly increased compared with those in the control group (Table 1, Fig. 2d and e). However, compared with the MCT + sham group, the RVSP and mPAP in the MCT + TCST group were significantly decreased (RVSP: 35.21 ± 5.59 mmHg vs. 48.36 ± 5.44 mmHg, mPAP: 22.02 ± 4.03 mmHg vs. 31.71 ± 2.94 mmHg, respectively; both *P* < 0.01; Table 1, Fig. 2d and e).

### Effects of TCST on RV hypertrophy and cardiac function in MCT-induced PAH

**Fig. 3:**
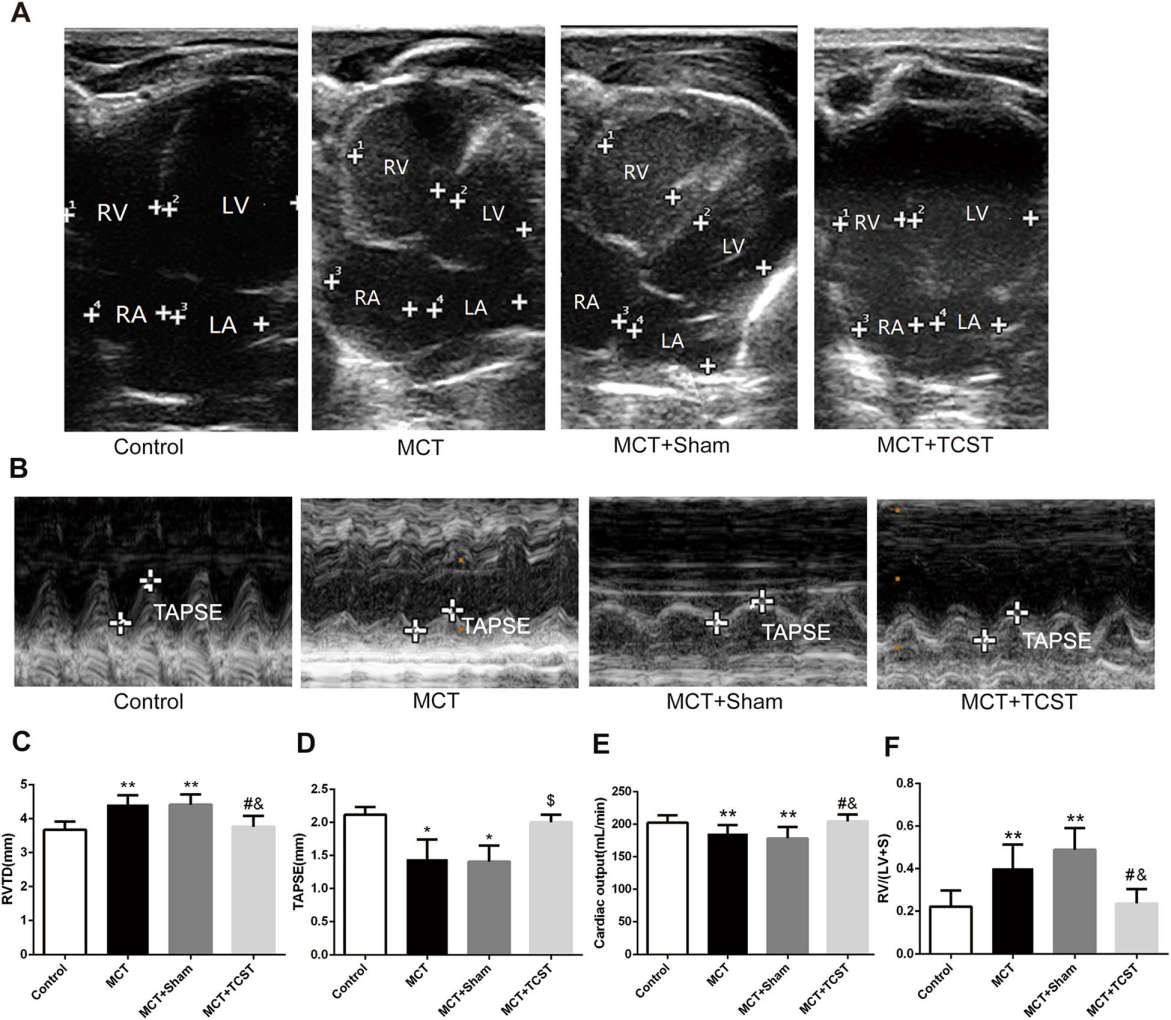
TCST improves right heart function and attenuates RV hypertrophy induced by MCT. **a** Representative echocardiography images of an apical 4-chamber view in the different groups. **b** Representative echocardiography M-mode images of TAPSE. **c**, **d** Quantitative analysis of the RVTD and TAPSE. **e** CO as assessed by echocardiographic measurements. **f** The ratio of the RV to RV/LV + S. **P < 0.01 vs. control group; #*P* < 0.01 vs. MCT group; &*P* < 0.01 vs. MCT + sham group; *P < 0.05 vs. control group; $*P* < 0.05 vs. MCT or MCT + sham group

The RVTD and RV/LV + S in the MCT + TCST group were significantly lower than those in the MCT + sham group (RVTD: 3.78 ± 0.40 mm vs. 4.36 ± 0.29 mm; RV/LV + S: 0.24 ± 0.07 vs. 0.48 ± 0.10; both *P* < 0.01; Table 1, Fig. 3a, c and f), suggesting that TCST decreased RV hypertrophy in the PAH rats. Additionally, compared with the MCT + sham group, the TAPSE and CO were significantly increased in the MCT + TCST group (TAPSE: 2.00 ± 0.12 mm vs. 1.41 ± 0.24 mm; *P* < 0.05; CO: 204.72 ± 11.13 ml/min vs.178.53 ± 6.75 ml/min; *P* < 0.01; Table 1, Fig. 3b, d and e), demonstrating that TCST improve the right ventricular function in PAH.

### TCST attenuated the medial thickening of the pulmonary artery induced by MCT

**Fig. 4:**
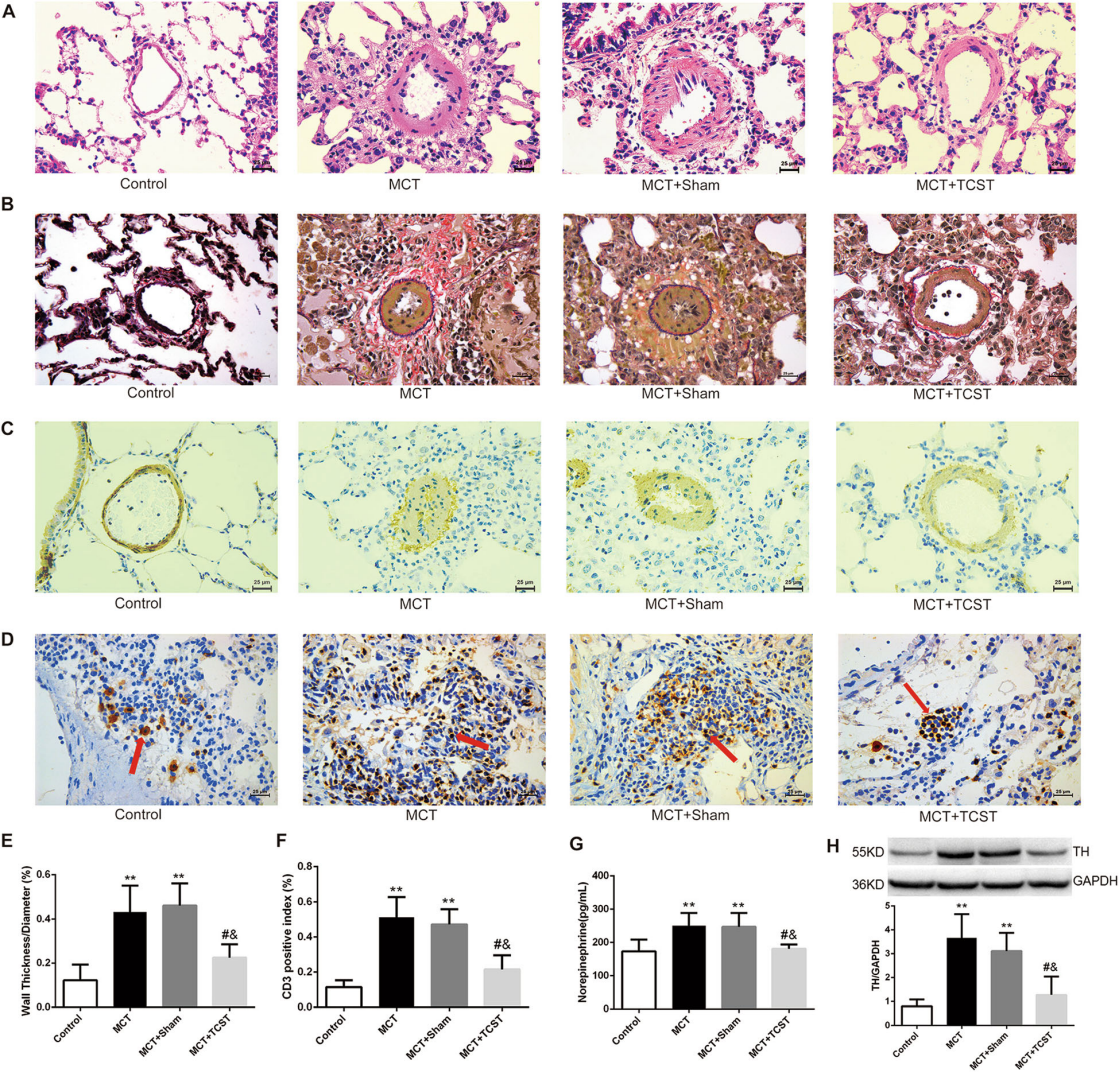
TCST attenuated pulmonary vascular remodelling induced by MCT. **a** Representative H & E stained pulmonary artery sections from each group of rats (scale bar = 25 μm). **b** Representative Elastin-Van Gieson (EVG) staining images of the pulmonary artery (scale bar = 25 μm). **c** Representative immunohistochemical staining images of α-SMA in the pulmonary artery (scale bar = 25 μm). **d** Representative photomicrographs of CD3 positive cells in the lung (red arrows indicate positive cells, scale bar = 25 μm). **e** The ratio of wall thickness (WT)/vessel radius in the pulmonary arteries. **f** Quantification of CD3 positive cells in each group. **g**, **h** TCST reduced the NE concentrations in lung homogenate and TH expression in the pulmonary artery tissues, which were significantly increased in the MCT rats. **P < 0.01 vs. control group; #*P* < 0.01 vs. MCT group; &*P* < 0.01 vs. MCT + sham group

To investigate the effects of TCST on pulmonary vascular remodelling in the MCT-induced PAH model, the morphology of the pulmonary vessels was examined by H & E, Elastic-Van Gieson and immunohistochemical staining of α-SMA. As shown in Fig. 4 (a, b, c, and e), the pulmonary artery WT% was greatly enhanced in the MCT group compared with that in the control group (40.21 ± 3.70% vs.12.20 ± 2.23%; P < 0.01). Nonetheless, the WT% was dramatically reduced in the TCST group compared to that in the MCT and MCT + sham groups (22.48 ± 1.75% vs. 40.21 ± 3.70% and 46.10 ± 3.16%; both P < 0.01). These results suggested that the rats with MCT-induced PAH exhibited exaggerated pulmonary vascular remodelling, but such remodelling was attenuated by TCST. In addition, to determine the effects of TCST on lung inflammation induced by MCT, the lung tissue sections were stained with a T-cell marker (CD3). The results showed that TCST significantly decreased MCT-induced T-cell accumulation (51.28 ± 3.48% in the MCT group vs. 21.56% ± 2.82% in the MCT + TCST group; P<0.01, Fig. 4d and f).

### TCST reduced the concentrations of NE in lung tissues of PAH rats

The concentrations of NE in lung tissues of MCT rats were significantly higher than that in the control group (250.5 ± 15.64 pg/mL vs. 173.6 ± 13.19 pg/mL, P<0.01). Whereas, the concentrations of NE in MCT + TCST rats decreased significantly compared with MCT + sham group (181.0 ± 4.83 pg/mL vs. 247.1 ± 16.91 pg/mL; *P*<0.01, Fig. 4g). Moreover, MCT significantly increased the expression of TH (the rate-limiting enzyme of NE production) compared with control group, which were inhibited by TCST treatment (Fig. 4h).

### TCST inhibited MCT-induced cell proliferation and increased cyclin protein expression in vivo

**Fig. 5:**
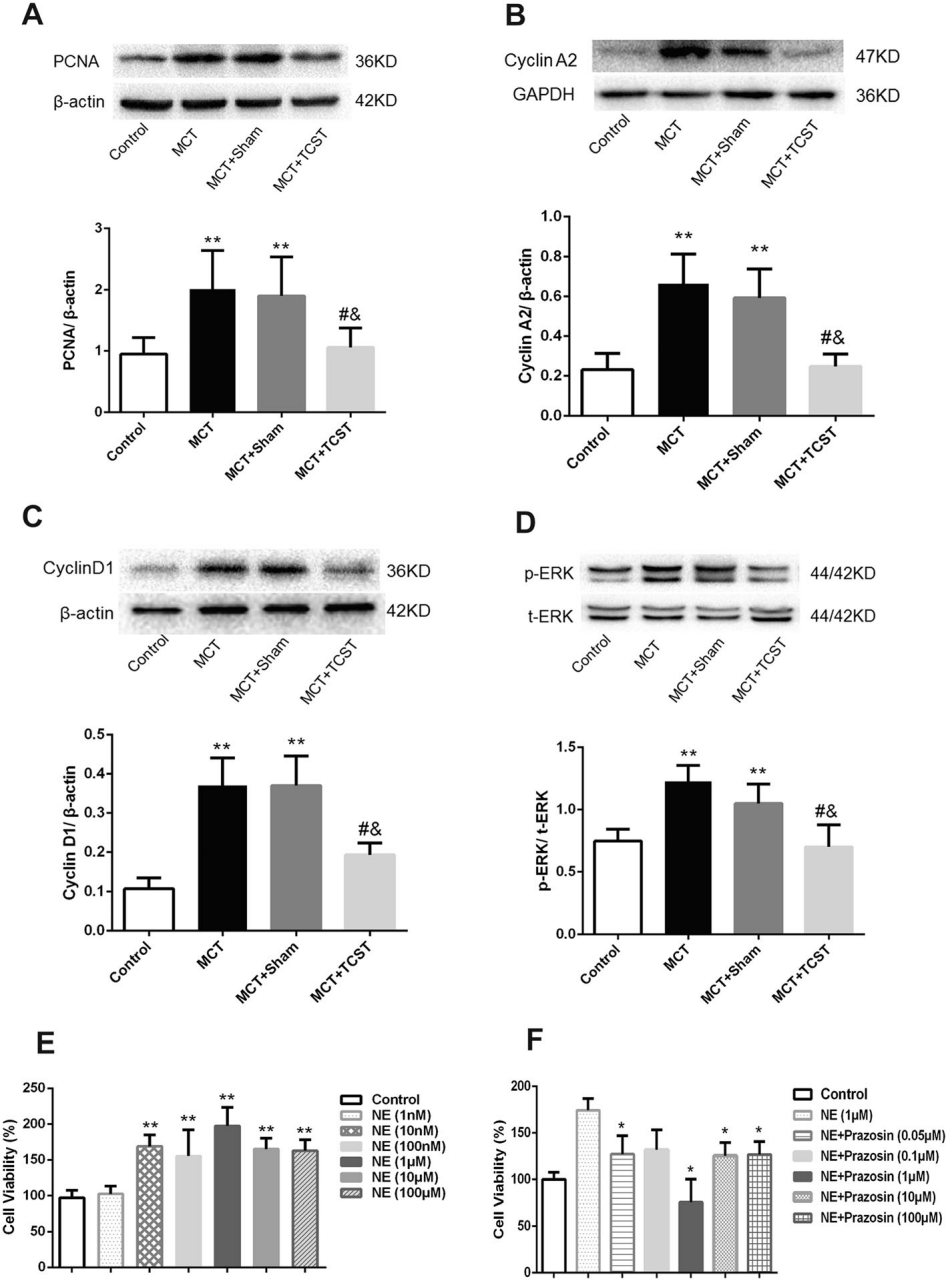
TCST attenuated MCT-induced proliferation and increased cyclin protein expression in vivo. **a** TCST decreased the upregulated expression of PCNA induced by MCT. **b**, **c** The upregulated expression of cyclin A2 and cyclin D1 induced by MCT was inhibited by TCST. **d** ERK activation in the pulmonary vasculature in the different groups. **P < 0.01 vs. control group; #*P* < 0.01 vs. MCT group; &*P* < 0.01 vs. MCT + sham group. **e** NE led to a significant increase in cell viability at a concentration of 1 μM. **P < 0.01 vs. control group; (**f**) Different concentrations of Pra inhibited the NE-induced increase in cell viability. *P < 0.05 vs. NE group

To determine whether cell proliferation is inhibited by TCST, we determined the protein expression of PCNA, cyclin A2 and cyclin D1 in pulmonary artery tissues. The data showed that compared with the control group**,** MCT significantly increased the expression of PCNA, cyclin A2 and cyclin D1 in the MCT-induced PAH rats, whereas the expression levels of these proteins were decreased in the MCT + TCST group (Fig. 5a, b, and c). Moreover, ERK-1/2 phosphorylation in the pulmonary artery tissues was significantly increased in the MCT group compared with that in the control group but was reduced in the MCT + TCST group (Fig. 5d). These results indicated that TCST may attenuate MCT-induced PAH partially by inhibiting the ERK-1/2 pathway.

### NE mediated proliferation in PASMCs

A CCK-8 assay was applied to determine the effect of NE on PASMC proliferation. A dose-dependent effect was observed by treating the PASMCs with 1 nM, 10 nM, 100 nM, 1 μM, 10 μM and 100 μM NE. These results show that NE caused a significant increase in PASMC viability at a concentration of 1 μM (Fig. 5e). Additionally, we investigated the effect of Pra on NE-induced cell proliferation. The PASMCs were treated with increasing doses (0.05 μM, 0.1 μM, 1 μM, 10 μM, and 100 μM) of Pra before the NE treatment. The results showed that Pra decreased pulmonary artery smooth muscle cell proliferation at a concentration of 1 μM (Fig. 5f). Based on our dose-response effects, we decided to use NE and Pra at 1-μM concentration in the subsequent experiments.

### Effect of ERK-1/2 on NE-induced proliferation in PASMCs

To further elucidate whether the ERK-1/2 pathway participates in NE-mediated proliferation, we blocked ERK-1/2 with U0126 and blocked NE with Pra.

**Fig. 6:**
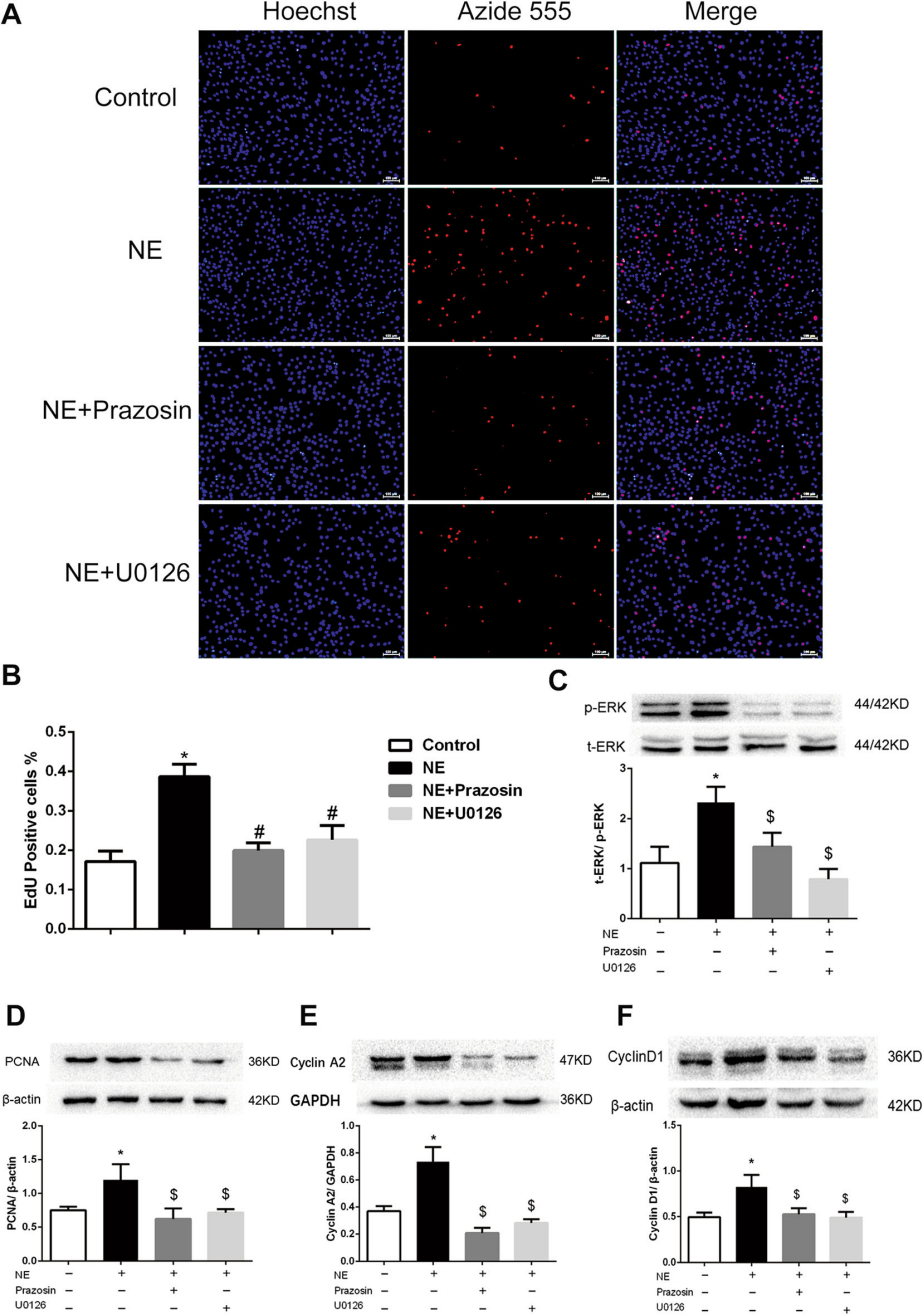
Effect of ERK-1/2 on NE-induced proliferation in PASMCs. **a** PASMC cell proliferation was measured by an EdU staining assay (scale bar = 100 μm). **b** NE increased the percentage of EdU-positive PASMCs. However, this effect was suppressed by the treatment with Pra or U0126. **c** NE increased ERK-1/2 phosphorylation in the PASMCs, and this increase was inhibited by Pra and U0126. **d**, **e**, **f** NE increased the expression of PCNA, cyclin A2 and cyclin D1 in the PASMCs, whereas Pra and U0126 attenuated these effects. *P < 0.05 vs. control group; $*P* < 0.05 vs. NE group

As shown in Fig. 6c, NE increased the phosphorylation of ERK-1/2, and this increase was reduced by Pra or U0126, suggesting that ERK-1/2 is likely involved in NE-mediated proliferation in PASMCs. In addition, Pra decreased proliferation by almost two-fold as measured by EdU in the NE-induced PASMCs, and U0126 had a similar effect (Fig. 6a and b). Furthermore, compared with the control group, NE promoted the expression of PCNA, cyclin A2 and cyclin D1 in the PASMCs, and this increase was suppressed by the Pra or U0126 treatment (Fig. 6d, e, and f).

## Discussion

The present study mainly demonstrated that TCST suppresses sympathetic activity by decreasing the NE concentrations in lung tissues, thereby attenuating pulmonary artery remodelling and RV hypertrophy in MCT-induced PAH rats. ERK activation associated with MCT was also found to be inhibited after TCST, and the inhibition of ERK activation reduced NE-induced proliferation in PASMCs. These findings may explain the link between PAH and excessive sympathetic activation, offering a potential therapy with sympathetic blocks in the treatment of PAH.

Anatomical studies have shown that the pulmonary vasculature is surrounded by adventitia, distributed sympathetic, parasympathetic, and sensory nerve fibres [26]. The adrenergic nerves surrounding the pulmonary arteries have been visualized with fluorescence histochemistry to detect catecholamines [27, 28], and the increased vascular resistance has been shown to be mediated by α-adrenoreceptors upon sympathetic nerve stimulation [29]. Moreover, sympathetic hyperactivity in PAH is evidenced by increased muscle sympathetic nerve activity and impaired HR variability [7], and this sympathetic hyperactivity has been reported to parallel the progression of PAH [8]. These findings form the theoretical basis of sympathetic blockade for the treatment of PAH. TCST had the distinct advantage of specific sympathetic denervation without adjacent parasympathetic nerve and vascular injury. Our previous studies have shown that the prognosis of rats with PAH was related to the TCST operation time, starting TCST earlier resulted in a better effect. Moreover, no significant change in the mPAP was found in control rats after TCST (Additional file 1). The present study showed that TCST attenuated the RVSP, mPAP, RV hypertrophy, and pulmonary vascular remodelling in the MCT-induced PAH rats. In addition, no negative effect on the HR and systemic blood pressure was observed in the experimental rats undergoing TCST. Na et al. reported that a left cervical ganglion block could be used to treat PAH by increasing the availability of nitric oxide [30], but there are several limitations to their results as follows: 1) the mPAP was not measured, 2) due to the predominance of the right stellate ganglion in the sympathetic control of the heart [31], it is better to block the right superior cervical ganglion than the left, and 3) according to our experience, it is too late to block the sympathetic nerve starting at day 14 after the MCT injection (Additional file 1).

TCST also inhibits RV remodelling. The RVTD, which is a mid-cavitary diameter measured on an apical 4-chamber view, is the most practical linear diameter used in clinical practice. The upper normal limit of the RVTD in humans is 35 mm [32]. The TAPSE is always used for the initial diagnosis of RV function due to its reliability and sensitivity. Among 63 patients with PAH, Forfia et al. found that for each 1-mm decrease in the TAPSE, the risk of death increased by 17% [33]. Similar results were obtained by Ghio et al. [34]. The results of the present study showed that the changes in the RVTD in each group of rats were consistent with the changes in the mPAP, and the change in the TAPSE was inversely proportional to the change in the mPAP.

A series of studies reported that the systemic sympathetic nervous system is activated in PAH patients and that NE results in adrenergic receptor-mediated vasoconstriction, dysregulated proliferation, and fibrosis through different signalling mechanisms [35]. Studies have shown that plasma venous NE levels are elevated and inversely correlated with CO in PAH patients [7, 36]. In the present study, we found that the concentrations of NE in lung tissues of MCT rats were significantly higher than those in the controls, leading to hypertrophy of the pulmonary artery, decreased CO. These findings were consistent with previous reports indicating that NE may aggravate heart failure and pulmonary artery remodelling [21, 37]. More importantly, the findings demonstrate that the decrease in the lumen area and the increase of the pulmonary arteries in MCT-induced PAH were inhibited by TCST. Furthermore, the accumulation of T cells was observed around the lung blood vessels in the MCT group, implying that MCT leads to inflammatory responses in the lung in rats. However, this phenomenon was significantly alleviated by TCST, indicating that TCST effectively inhibited MCT-induced lung inflammation. One explanation could be that TCST reduced the concentration of NE, which binds adrenergic receptors on immune cells in the PAH rats, inhibiting the consequent response [38]. These findings support the hypothesis that TCST can reduce the release of NE and inhibit PAH progression.

MCT is a toxic alkaloid that can induce PAH via endothelial damage and PASMC proliferation. The proliferation of PASMCs has been closely linked to pulmonary arterial remodelling, which is the main feature of PAH. In addition, the cell cycle plays a central role in cell proliferation and consists of the following distinct stages: G1, S, G2 and M phases; the progression of the cell cycle is precisely controlled and coordinated by a series of cyclins. Cyclin D1, which is expressed in numerous tissues, is a critical target of proliferative signals during the G1 phase and plays a crucial role in the G1/S transition [39]. The overexpression of cyclin D1 is known to be positively associated with the pulmonary vessel WT in rats and is correlated with the early onset of cancer [40, 41]. Studies have shown that cyclin A2 is a target of oncogenic signals; in complex with cdk1, cyclin A2 promotes entry into mitosis [42]. In addition, the upregulated expression of cyclin A2 leads to PASMC proliferation [43]. The results of the present study show that MCT increased the expression of cyclin D1 and cyclin A2 in vivo, suggesting that MCT promoted cell cycle progression. However, these trends were inhibited by TCST.

We further explored the mechanism of action of NE in the process of PASMCs proliferation. ERK-1/2 is a member of the MAPK family that plays an important role in multiple cellular functions, including growth and differentiation. Previous studies have indicated that increasing proliferation in PASMCs is a part of the sustained activation of ERK-1/2 and that the inhibition of ERK-1/2 activation may be a therapeutic target in PAH [21, 43, 44]. Based on the results of the present study, the phosphorylation of ERK-1/2 was significantly increased in the pulmonary arteries of the MCT-treated rats, which was consistent with the change in NE. In vitro, NE promoted PASMC proliferation at the optimal concentration of 1 μM and increased the phosphorylation of ERK-1/2 and proliferation markers, such as PCNA, cyclin D1 and cyclin A2. Moreover, ERK-1/2 phosphorylation was abolished by the treatment with Pra, and the expression of the proliferation markers was prevented by U0126 (a specific inhibitor of ERK-1/2). These results suggest that NE facilitates PASMC proliferation partially via the activation of the ERK-1/2 signalling pathway.

Sympathetic activation may be a compensatory response that improves cardiac function during the early stages, but marked sympathetic hyperactivity aggravates the progression of heart failure. In addition, RV failure is one of the pathologies of PAH, and reduced parasympathetic nervous activation is associated with disease progression in PAH [45]. Although some studies suggest that drugs that depress sympathetic nervous activity, such as adrenergic antagonists, could be used to treat PAH, the value of vasodilatory treatment in PAH is still controversial [21, 46, 47]. First, because α1-adrenergic receptors are expressed in pulmonary vascular tissue at density levels similar to resistance blood vessels, most α-adrenergic antagonists usually result in systemic hypotension. Early α-adrenergic antagonists were used to treat PAH included prazosin and phentolamine; however, the clinical application of such drugs is restricted due to their side effects, including dyspnoea, systemic hypotension, and adverse haemodynamic effects [48, 49]. Second, β-adrenergic antagonists have recently been considered a therapeutic strategy for PAH, and some small observational studies have reported that 90% of patients demonstrated significant improvement in the 6-min walk distance test following β-blocker treatment [50]. However, some experts have reservations regarding the safety profile of β-blockers. A clinical study reported that bisoprolol produced no improvement in the RV ejection fraction but resulted in a decrease in both the HR and cardiac index and exhibited a trend towards reduced exercise capacity [51]. The present study demonstrated that TCST improved the haemodynamics, pulmonary artery remodelling, and RV function in MCT-induced PAH rats. Additionally, one advantage of TCST is that this treatment has no effect on systemic blood pressure, HR or CO. Thus, adrenergic antagonists may not be an alternative for TCST.

This study has several limitations. First, sympathetic reinnervation has been shown to occur in a previously denervated rat lung [52]. However, Horner’s syndrome in rats did not disappear during our study, indicating that the sympathetic reinnervation was not complete. Second, there was an absence of histological evidence of pulmonary sympathetic nerves, demonstrating a lack of histological proof of sympathetic nerve changes after TCST. Third, whether the other two signalling pathways of the MAPK family, i.e., JNK and P38, are also involved in mediating the TCST-induced attenuation of the progression of PAH was not determined.

## Conclusion

The present study demonstrates that TCST is an effective and specific method of sympathetic denervation that does not affect systemic blood pressure or cause injury to the blood vessels and parasympathetic nerves. Moreover, TCST inhibited the MCT-induced progression of PAH in rats likely by decreasing the NE concentrations in the lung tissues and thereby inhibiting PASMCs proliferation mediated by the ERK-1/2 signalling pathway. We provided novel insight into the use of this nonpharmacologic strategy in patients with PAH.

## Additional file

Additional file 1: Figure legends (A) To determine the optimal time to intervene, we have conducted a preliminary experiment. Different time-point of TCST were checked and compared in the MCT rats as follows: the same day (D0), the third day (D3), the seventh day (D7) and the fourteenth day (D14) after MCT injection, respectively. Then the pulmonary arterial pressure was measured by right heart catheterization on 21 day (D21) after MCT administration. (B) Our results found the rats with lowest mPAP was that undergoing TCST on the same day of MCT injection among treatment groups. But no significant decrease of mPAP was found in rats when the TCST time started on 14th day. Besides, there was no significant change in mPAP in control +TCST group. As a result, the prognosis of rats with PAH was related with TCST operational time, the earlier TCST started, the better effect was. * *P* < 0.01 vs. control group; # P < 0.01 vs. MCT group. (C) Cardiac output (CO) was measured on M-mode echocardiography parasternal long-axis views. (D) Pulmonary artery transverse diameter (PATD) were measured on parasternal short-axis views. (E) Right ventricular transverse diameter (RVTD) were measured on apical 4-chamber view. AO, aorta; LA, left atrium; LV, left ventricle; PA, pulmonary artery; RV, right ventricle. (TIF 9101 kb)

## Data Availability

The datasets used and/or analysed during the current study are available from the corresponding author upon reasonable request.

## Abbreviations

CO: Cardiac output
ERK: Extracellular signal-regulated kinase
HR: Heart rate
IP: Intraperitoneal injection
LV: Left ventricle
MCT: Monocrotaline
mPAP: Mean pulmonary arterial pressure
mSAP: Mean systemic arterial pressure
NE: Norepinephrine
PADN: Pulmonary artery denervation
PAH: Pulmonary arterial hypertension
PASMCs: Pulmonary arterial smooth muscle cells
PATD: Pulmonary artery transverse diameter
PCNA: Proliferating cell nuclear antigen
Pra: Prazosin
RV: Right ventricular
RVSP: Right ventricular systolic pressure
RVTD: Right ventricular transverse diameter
S: Septum
SGBs: Stellate ganglion blocks
TAPSE: Tricuspid annular plane systolic excursion
TCST: Transection of the cervical sympathetic trunk
TH: Tyrosine hydroxylase
WT: Medial wall thickness
α-SMA: α-smooth muscle actin
