# Supplementary figures and images for: Transection of the cervical sympathetic trunk inhibits the progression of pulmonary arterial hypertension via ERK-1/2 Signalling
Source: Respir Res. 2019 Jun 14;20:121. doi: 10.1186/s12931-019-1090-2 (PMC6567667; doi:10.1186/s12931-019-1090-2)

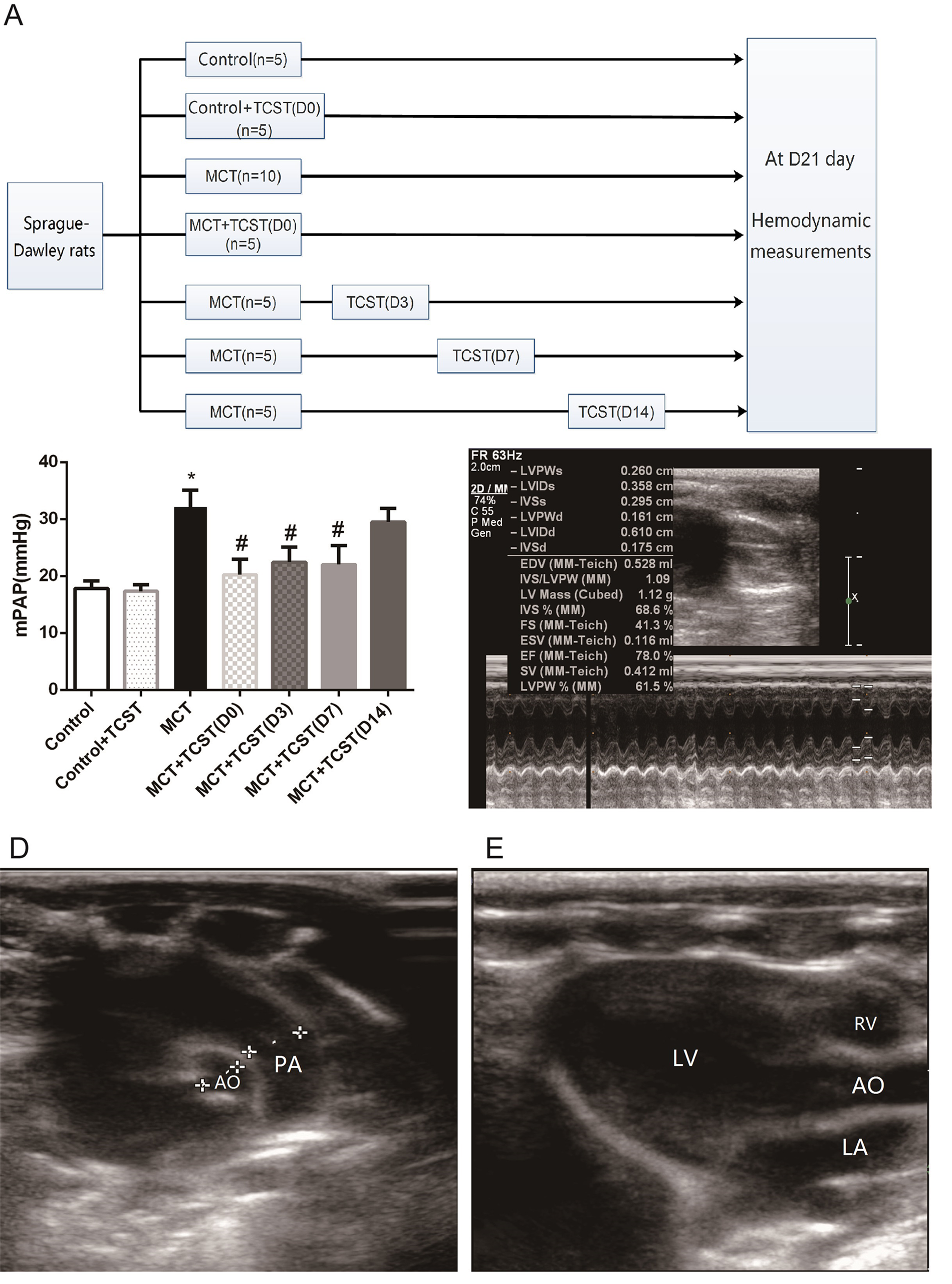

Supplement: Supplementary file 1 — Figure legends (A) To determine the optimal time to intervene, we have conducted a preliminary experiment. Different time-point of TCST were checked and compared in the MCT rats as follows: the same day (D0), the third day (D3), the seventh day (D7) and the fourteenth day (D14) after MCT injection, respectively. Then the pulmonary arterial pressure was measured by right heart catheterization on 21 day (D21) after MCT administration. (B) Our results found the rats with lowest mPAP was that undergoing TCST on the same day of MCT injection among treatment groups. But no significant decrease of mPAP was found in rats when the TCST time started on 14th day. Besides, there was no significant change in mPAP in control +TCST group. As a result, the prognosis of rats with PAH was related with TCST operational time, the earlier TCST started, the better effect was. * P < 0.01 vs. control group; # P < 0.01 vs. MCT group. (C) Cardiac output (CO) was measured on M-mode echocardiography parasternal long-axis views. (D) Pulmonary artery transverse diameter (PATD) were measured on parasternal short-axis views. (E) Right ventricular transverse diameter (RVTD) were measured on apical 4-chamber view. AO, aorta; LA, left atrium; LV, left ventricle; PA, pulmonary artery; RV, right ventricle. (TIF 9101 kb) [file 12931_2019_1090_MOESM1_ESM.tif]
